# Connectomes in Temporal Lobe and Idiopathic Generalized Epilepsies

**DOI:** 10.3390/jcm14113744

**Published:** 2025-05-27

**Authors:** Lukas Machegger, Pilar Bosque Varela, Bernardo Crespo Pimentel, Giorgi Kuchukhidze

**Affiliations:** 1 Department of Neuroradiology, Christian Doppler University Hospital, Paracelsus Medical University, 5020 Salzburg, Austria; l.machegger@salk.at; 2 Department of Neurology, Neurocritical Care and Neurorehabilitation, Christian Doppler University Hospital, Centre for Cognitive Neuroscience, Member of the European Reference Network EpiCARE, Paracelsus Medical University of Salzburg, 5020 Salzburg, Austria; pi.bosque-varela@salk.at (P.B.V.); bernardo.pimentel@gesundheitsverbund.at (B.C.P.); 3 Department of Neurology, Hietzing Hospital and Neurological Center Rosenhügel, 1130 Vienna, Austria; 4 Neuroscience Institute, Christian Doppler University Hospital, Centre for Cognitive Neuroscience, 5020 Salzburg, Austria; 5 Karl Landsteiner Institute for Neurorehabilitation and Space Neurology, 5020 Salzburg, Austria

**Keywords:** Connectome, MRI, epilepsy

## Abstract

Epilepsy is widely known as a network disease. Ictal and interictal activities are generated and spread within the existing networks involving different regions of the brain. Network alterations affect both grey and white matter, deep brain nuclei, including those of the ascending reticular formation. These structures may be involved in a disorganized connectome associated with epilepsy. A growing body of neuroimaging and neuropsychological findings suggests that global and focal network aberrations are closely linked to cognitive deficits in epilepsy patients. This evidence relates equally to focal epilepsies, such as temporal lobe epilepsy or extra-temporal lobe epilepsy, as well as generalized epilepsies, such as juvenile myoclonic epilepsy. Network abnormalities have been associated with a broad range of cognitive impairments, including language, memory, and executive functions, as well as sensory and motor functions. Whole-brain structural connectome models help in the understanding of seizure generation and spread. Identifying key nodes of seizure propagation may help in planning surgical procedures in individual patients by simulating epilepsy surgery on virtual models. Functional connectomic profiles may predict seizure outcomes in patients who undergo deep brain stimulation due to intractable seizures. Therefore, individualized interventional strategies could be developed based on connectome characteristics.

## 1. Introduction

The human brain represents a sophisticated network of interconnected neurons [1]. This network contains hubs—clusters of neurons heavily connected with multiple regions and modules—with a highly specific function. Hubs are essential for the transfer of information, and modules for specific cognitive processes [1,2]. There are both structural and functional brain networks (Table 1). In structural networks, the physical connections between the brain regions may be depicted by a diffusion tensor imaging (DTI) sequence of MRI [3,4,5]. To measure the connectome, high-resolution gray matter images are normalized to the diffusion space and divided into regions of interest (ROIs), which may be defined using brain atlases or as individual voxels. The fiber count connecting pairs of ROIs can be organized into a weighted matrix, representing the structural connectome [6]. This count can be adjusted for factors such as the distance between ROIs and their respective volumes. Additionally, setting a fiber count threshold can simplify the connectivity matrix, focusing on significant links [6,7,8,9]. While diffusion MRI focuses on white matter pathways, covariance analysis of morphological markers compares gray matter distributions among individuals. This approach, though correlational, has been increasingly applied to individual-level analyses, helping map networks based on morphological and microstructural similarities. By identifying networks with similar cytoarchitecture, this method distinguishes between sensory–motor regions and association areas with more extensive connections [6].

Functional networks act synchronously during specific cognitive tasks or even during the resting state [3,5].

Functional connectomes focus on assessing the activity of interconnected gray matter rather than the physical connections of the brain. Functional connectivity is derived from the low-frequency blood oxygen level-dependent (BOLD) signals captured, for instance, during resting-state fMRI. Regions that show synchronous fluctuations are considered functionally linked, even without direct structural connections [8].

An extension of this concept is effective connectivity, which measures how activation in one ROI influences the activation in others [9]. This approach takes into account the time- and task-dependent nature of neural activity, enabling the exploration of causality within brain functions. Effective connectivity is not limited to fMRI; it can also be assessed using techniques with superior temporal resolution, such as magnetoencephalography or high-density EEG, or a combination of these methods. This capability is particularly promising for advancing our understanding of the dynamics between structure and function in networks related to conditions like epilepsy [9].

Brain networks are prone to rewiring due to their plasticity in both health and disease [10]. Brain connectomes refer to comprehensive maps of neural networks.

In recent years, an enormous body of literature has been published in relation to brain connectomes, and different connectome databases have been developed [11,12]. Studying physiological and aberrant brain networks has been instrumental in understanding the communication of various brain regions during complex cognitive processes.

In epilepsy research, investigating neural connectomes allows a better understanding of changing neural connectivity patterns during seizure and the interictal period. Research related to brain connectomes helps in understanding the ways of seizure initiation and propagation, as well as in defining potential biomarkers for seizure prediction and outcome.

Connectomes can also guide treatment strategies in patients with drug-resistant seizures in terms of targeting epileptic networks by neuromodulation techniques such as deep brain stimulation (DBS) [13]. Connectomes may also assist in the development of new drugs that aim at the key nodes and pathways involved in epileptogenesis. Thus, understanding unique individual brain connectivity may pave the way for individualized treatments in epilepsy patients by addressing specific aberrations in their connectomes.

## 2. Understanding the Mechanisms of Epileptogenesis

Epilepsy is widely known as a network disease. Ictal and interictal activities are generated and spread within the existing networks involving different regions of the brain [14]. The evidence on brain connectomes affected in epilepsy comes from neurophysiological and neuroimaging studies showing aberrant networks both in focal and idiopathic genetic generalized epilepsies.

One of the most well-studied focal epilepsy syndromes is temporal lobe epilepsy (TLE). Abnormal early neurodevelopment and atypical brain aging in TLE have been linked to subtle changes in temporal lobe cortical architecture, cortical folding, aberrant cortical/subcortical interface, hippocampal malrotation, and eventually abnormal connectivity within the entire limbic system [15,16]. Network alterations progress over time in TLE, reflecting the influence of seizure frequency, effects of medications, and psycho-social factors [17].

In unilateral drug-resistant TLE vs. healthy controls, TLE showed ipsilateral thalamic atrophy, including its anterior, medial, and posterior divisions. Thalamic atrophy was associated with bilateral thinning of fronto-central and lateral and mesial temporal cortices, as well as with longer duration of epilepsy [18].

Abnormal local connectivity and diminished long-range projections could result in network segregation, a structural configuration that promotes recurrent seizure activity in TLE. Utilizing a novel in vivo technique that integrated resting-state functional connectivity with the physical distances between cortical regions, Lariviere et al. quantified the relationship between short- and long-range functional connections [19]. When comparing drug-resistant TLE patients to control subjects, significant decreases in connectivity within the temporo-limbic and dorsomedial prefrontal cortices were observed. These connectivity reductions appeared to be influenced by simultaneous increases in short-range connections and decreases in long-range connections in TLE patients. Furthermore, when examining associated morphological and microstructural changes, it was found that the connectivity reductions were not linked to atrophy in the temporal or fronto-central cortices; rather, they were associated with alterations in the superficial white matter originating from the mesio-temporal region. Additionally, a supervised machine learning algorithm was able to identify features related to connectivity distance that predicted the likelihood of seizure recurrence after surgery with 76% accuracy, outperforming models based solely on clinical data and hippocampal MRI characteristics, indicating potential prognostic value [19].

In another study focusing on a selective, edge-wise method, which analyses the distribution of white matter pathways associated with a node, all aberrant cross-hemispheric connections were associated with focal-to-bilateral tonic–clonic seizures [20]. Most of the abnormalities were located in the opposite hemisphere, with graph metric values generally lower compared to healthy controls. The contralateral amygdala exhibited significant reductions in structural connection pathways to the corresponding frontal lobe. In TLE, abnormal connections were associated with the amygdala, where the ipsilateral side showed increases in connectivity, while the contralateral side showed a decrease. Connectivity effects observed in patients with focal-to-bilateral tonic–clonic seizures were influenced by factors such as age, recent seizure frequency, and the duration of the illness [20].

Excitation–inhibition imbalance is thought to be a crucial mechanism in the pathophysiology of epileptogenesis. The Hurst exponent, which serves as an index of the excitation–inhibition ratio, can be calculated from resting-state fMRI time series demonstrating functional connectivity in TLE patients [21]. A notable decrease in the Hurst exponent was linked with pharmacoresistance, indicating more excitable network dynamics. Connectome decoders identified the temporo-limbic and fronto-central cortices as potential network epicenters for excitation–inhibition imbalance. Additionally, computational simulations show that elevated cortical excitability in TLE is associated with abnormal increases in the strength of recurrent connections among local neuronal ensembles.

TLE patients with longer disease duration, more frequent electroclinical seizures, interictal epileptic spikes, and poorer cognitive functioning exhibited a more pronounced elevation in the excitation–inhibition ratio [21].

As opposed to TLE, in patients with idiopathic generalized epilepsies (IGE), more global and widespread network aberrations have been observed in advanced neuroimaging studies. The core of network irregularities is in thalamo-cortical connections, which may influence mechanisms of epileptogenesis in both TLE and IGE.

In a multimodal imaging study on 107 TLE and 96 IGE patients as well as 65 healthy controls, structural and functional network analyses revealed considerable atrophy, microstructural disruptions, and a reduction in thalamo-cortical connectivity in TLE patients [22]. In contrast, IGE patients exhibited only minor structural abnormalities alongside increased thalamo-cortical connectivity. Additionally, connectome-informed biophysical simulations showed that IGE was associated with slight increases in subcortical drive that contributed to cortical dynamics. Conversely, TLE was characterized by diminished subcortical drive and an imbalance between excitation and inhibition within limbic and somato-motor microcircuits. This network analysis enables distinguishing between TLE and IGE at the system level, showing paradoxically more pronounced imbalances in microcircuits and macroscale networks in focal compared to generalized epilepsy [22].

Fronto-mesial, para-ventral, and para-limbic cortices have also been implicated in atypical connectomes related to IGE [23]. A certain level of focality has been observed in IGE patients in generators of epileptiform discharges [24], as well as in resting states [25].

A large study, ENIGMA-Epilepsy (Enhancing NeuroImaging Genetics through Meta-Analysis), conducted in multiple centers worldwide, analyzed structural covariance networks in patients with epilepsy and related findings to post-mortem epilepsy risk gene expression patterns [26]. Graph theoretical analysis on MRI-based cortical thickness and subcortical volume correlations was performed on 578 patients with TLE: 288 with IGE, and 1328 healthy controls [26]. Patients with TLE, as opposed to healthy controls, had marked changes in a regional subnetwork of brain areas comprising orbito-frontal, temporal, and angular cortices in terms of increased clustering and path length. These alterations were observed in both right- and left-sided TLE. However, the changes were more prominent in left fronto-temporal cortices, probably due to asymmetrical damage of temporal lobes and higher connectivity of the dominant hemisphere [26]. Network aberrations in TLE were suggestive of more regularized, “lattice-like” subnetwork organization. In IGE, however, compared to healthy controls, the regional network analysis demonstrated decreased clustering and path length predominantly in fronto-temporo-parietal cortices bilaterally, suggesting a more randomized network organization as opposed to TLE [26].

Genetic factors have been closely linked with covariance network organization. In the ENIGMA study, spatial correlations between network profiles of TLE and IGE and epilepsy-related gene expression maps were assessed [26]. Significant associations between spatial patterns of multivariate network alterations in TLE and epilepsy risk gene expression levels of hippocampal sclerosis were observed. Network changes in IGE were also linked to the expression levels of genes associated with generalized epilepsies. These observations shed more light on the pathophysiology of IGE and TLE, indicating spatial correspondence between changes in network organizations and molecular phenotypes [26].

The heritability of hippocampal microstructural and functional connectivity has been studied within the Human Connectome Project in healthy adults [12]. It has been shown that connectivity between the microstructure of distinct sub-regions of the hippocampus, such as the subiculum, cornu ammonis, dentate gyrus, and isocortex, was genetically determined [27]. This connectivity consistently followed the heritable anterior–posterior axis: anterior hippocampal sub-regions connected largely to anterior frontal and temporal cortices, and posterior sub-regions to inferior temporal and visual cortical areas. In terms of intrinsic hippocampal axes, it was observed that lateral–posterior regions of the hippocampus were highly coupled, whereas anterior–medial subfields were uncoupled [27].

Heritability of functional organization was weaker than microstructural connectivity, most probably driven by individual experiences. This difference between the heritability of structure and function demonstrates an interplay between stability and plasticity, unique features of hippocampal formation [27].

## 3. Predicting Seizure Outcome

TLE is the most common drug-resistant epilepsy syndrome [28]. Most patients with TLE are effectively treated with anti-seizure medications (ASM); however, about a third of them develop pharmacoresistant seizures, which are potentially treatable by neurosurgical interventions [29]. Epilepsy surgery renders about 65% of patients seizure-free at 1 year [30,31]. However, the proportion of patients experiencing post-surgical seizures increases with time: at 2 years following epilepsy surgery, around 40% of patients experience seizures, and at 10 years, almost half of them have disabling seizures [32]. Multiple factors, such as strictly unilateral epileptiform discharges and hippocampal sclerosis, duration of epilepsy, age at epilepsy surgery, history of febrile seizures, extension of resection, etc., have been associated with a post-surgical outcome. However, none of them could reliably predict post-surgical seizure outcome in drug-resistant TLE.

Preoperative quantitative MRI techniques assessing structural and functional brain connectivity serve as promising novel prognostic biomarkers. Connectome fingerprints—personalized imaging biomarkers of seizure outcome—play an increasing role in the planning of epilepsy surgery in individual patients with drug-resistant TLE [33].

First, connectome studies in TLE were focused on the role of hippocampal sclerosis in an epileptic network. Diffusion tractography, graph theory, and novel network control theory paradigms were utilized for the generation of whole-brain connectomes [34]. It has been demonstrated that neuronal loss in the cornu ammonis (CA) 1–3 areas has an important impact on whole-brain connectomes. As opposed to hippocampal sclerosis, isolated gliosis of the hippocampus had minimal effects on the structural network alterations in TLE patients [34]. An aberrant structural network due to hippocampal sclerosis contributes to cognitive dysfunction, as has been shown by connectome-informed dynamic communication models [35].

Preoperative connectome data of nine patients with drug-resistant unilateral TLE who underwent epilepsy surgery and were seizure-free post-operatively were used to create a fingerprint of a favorable seizure outcome (Engel I) [33]. The connectome data was based on both structural and functional whole-brain connections. Thirty-eight other similar patients in the study with different post-surgical seizure outcomes were compared to the connectome fingerprint of the favorable seizure outcome [33]. Distance to fingerprint was significantly higher in patients with unfavorable seizure outcomes (Engel III-IV) compared to those with Engel I-II outcomes one year following epilepsy surgery. Receiver operating characteristic (ROC) analysis revealed 100% sensitivity and 90% specificity in identifying patients with an unfavorable seizure outcome. Distance to fingerprint was not related to other imaging, clinical, or EEG parameters such as age at MRI scan, duration of epilepsy, seizure frequency, presence of hippocampal sclerosis, lateralizing ictal or interictal EEG, or the results of FDG-PET [33]. The data of this study should be, however, interpreted cautiously, as the fingerprint was based on imaging data of a limited number of patients, and the outcome was assessed solely 1 year following epilepsy surgery [36]. Larger multicenter studies with longer outcome periods are necessary for obtaining more robust and replicable results [36].

In a study on 126 drug-resistant TLE patients and 60 healthy controls, two models were utilized: a data-driven method, which analyzes nodes and neighboring atrophy ranking, and a network diffusion model, which simulates the spread of aberrant connections from various seed areas [37]. This study revealed that structural, rather than functional, connectivity influences neuronal loss in TLE. Structural connectivity was considerably variable when analyzed with patient-specific modelling. It demonstrated, however, that the hippocampus and adjacent temporo-limbic structures were the epicenter for atrophy. Importantly, seizure freedom was associated with the localization of atrophy epicenters strictly in the ipsilateral temporal pole and mesial temporal area, whereas those patients with atrophy in the fronto-central regions had unfavorable seizure outcomes. These findings are in line with the concept of temporal lobe plus epilepsy with worse seizure outcomes as opposed to clear-cut mesial TLE [37].

Pre-surgical brain network alterations related to white matter tracts in epilepsy patients were also associated with post-surgical seizure outcomes. In a multicenter study on TLE patients, a machine learning technique was applied to betweenness centrality, a measure of network hubness. It assisted in showing that measuring node hubness and network integration may serve as reliable predictors of seizure freedom after epilepsy surgery. The nodes that were strongly associated with seizure freedom included mesial and lateral temporal areas, both ipsi- and contralateral to the seizure focus [38].

Another study analyzing the white matter tract network on pre-surgical diffusion-weighted images of 35 TLE patients also suggested that connectome hubs, which contributed to the accuracy of predicting post-surgical seizure freedom, involved temporal and extra-temporal regions of both hemispheres. In this study, a structural connectome model could predict with high precision both post-surgical seizure freedom (positive predictive value of 88%) and failure of seizure control (negative predictive value of 79%) [39].

The field of connectome-associated biomarkers of seizure outcomes is developing rapidly, and the findings are of importance, however, their use in routine clinical practice is still limited. The majority of studies include a limited number of patients, are monocentric, utilize diverse methodologies, lack long-term observation periods, cohort homogeneity, or multicenter validation [40,41,42,43]. Clinical translation of connectome-related predicting factors may be achieved by multicenter studies with homogeneous populations of patients, external validations, and uniform methodology.

## 4. Cognitive Deficits

A growing body of neuroimaging and neuropsychological findings suggests that global and focal network aberrations are closely linked to cognitive deficits in epilepsy patients. This evidence relates equally to focal epilepsies, such as TLE or extra-TLE, and generalized epilepsies such as juvenile myoclonic epilepsy.

TLE has been associated with a broad range of cognitive impairments, including language, memory, and executive functions, as well as sensory and motor functions.

The extent of white matter network aberrations may contribute to cognitive problems in TLE patients [44]. Disrupted cerebral intercommunication negatively affects cognition, especially in left TLE [44]. Structural connectomes can more reliably predict verbal memory impairment in drug-resistant TLE patients compared to hippocampal volumetry or clinical features [45]. Short-range temporo-temporal connections are important contributors to memory performance [45].

Structural connectomes can also serve as important biomarkers in predicting language disability in TLE patients [46]. Based on the affected white matter network, one can discriminate between patients with and without language impairment. Multiple bilateral and interhemispheric white matter connections contribute to language function; however, the most important network includes connections between the left superior temporal gyrus and frontal operculum [46].

In drug-resistant TLE patients, cognitive dysfunction in multiple domains, such as sensorimotor, attention, verbal fluency, and visuo-constructional ability, as well as memory, is related to aberrant hierarchical cortical organization [47]. Stepwise functional connectivity analysis revealed bidirectional disruptions of sensory–paralimbic functional organization in TLE patients as compared to healthy controls. Cognitive impairments were more pronounced in patients with a longer duration of epilepsy and a favorable seizure outcome following epilepsy surgery [47].

Cognitive dysfunction in TLE patients has also been associated with disorganization in brainstem arousal centers [48]. A reduction in the structural and functional connectivity of ascending reticular activating systems with neocortical regions was observed in TLE patients as opposed to healthy controls. Diminished structural connections between the cuneiform–subcuneiform nuclei and cortex were related to low IQ performance and reduced visuo-spatial memory, whereas less functional connectivity was linked to impaired verbal IQ and language abilities. A decrease in functional connectivity of brainstem arousal centers and the neocortex was associated with the presence of seizures with impaired consciousness and seizure generalization [48]. Aside from cuneiform–subcuneiform nuclei, aberrant connections of other structures of the ascending reticular activating system (dorsal raphe, locus coeruleus, median raphe, parabrachial complex, pontine oralis, and the pedunculopontine and ventral tegmental areas) to the neocortex also contribute to cognitive dysfunction in TLE patients [49].

The association of cognitive impairment and aberrant connectivity of white matter in drug-resistant TLE seems to be robust and reproducible across different centers [50]. In a multisite cohort of 95 TLE patients, reduced functional differentiation between sensory–motor networks and transmodal systems, such as the default mode network, with peak alterations in bilateral temporal and ventromedial prefrontal cortices, was observed in comparison to healthy controls [50]. This functional reorganization of white matter microarchitecture in temporo-limbic areas was independent of diffuse and bilateral TLE-related changes in cortical grey matter thickness. In this cohort of TLE patients, memory deficits were strongly associated with connectome alterations [50].

Novel approaches have been used for further exploration of the association between cognitive deficits and aberrant brain connectivity. Cortex-wide microstructural gradients were compared between 21 patients and 35 healthy controls in a study utilizing the generation of microstructural gradients [11]. A reorganization of this gradient was observed in TLE, driven by reduced microstructural differentiation between paralimbic cortices and the remaining cortex, with marked abnormalities in ipsilateral temporo-polar and dorsolateral prefrontal regions [11]. Similar topographic variations of cortical cytoarchitecture were observed on an independent post-mortem cohort, underlying the robustness of the in vivo findings. Furthermore, microstructural changes were associated with cognitive network reorganization shown during an episodic memory functional MRI paradigm and correlated with inter-individual differences in task accuracy [11].

Alterations in connectomes in TLE may be progressive and relate to both normative and non-normative, highly-individualized brain networks [51]. In a retrospective cross-sectional study on 100 TLE patients, it was demonstrated that cognitive deficits in multiple domains were associated with disruptions in canonical, normative networks, such as dorsal attention, default mode, and fronto-parietal networks. These aberrations could explain the inability of brain systems to cognitively compensate. As opposed to the cognitively impaired patients, the normative networks were intact in those without cognitive deficits. The non-normative, highly specific networks, however, were reorganized even in cognitively normal patients, suggesting that these alterations may predict disruptions in cognitive processes even before the changes in normative connectomes. The increased presence of non-normative networks in cognitively impaired patients may serve in both adaptive and maladaptive ways. Their frequent appearance in the tonic resting state is probably associated with an attempt to restore, for instance, an impaired language network. Dynamic connectome alterations in TLE demonstrate their association with a transition from normal to cognitive-impaired states [51].

Dynamic compensation changes in the language network affect different trajectories in TLE patients. Both left and right TLE patients demonstrated a reduction in intra-subsystem communication in the “core” left frontal language area. On the other hand, the left frontal area in left-TLE patients showed increased connections with the contralateral temporal so-called “peripheral” language system, indicating compensating recruitment of the healthy hemisphere [52].

Altered brain function in TLE, including cognitive domains, is associated with increased energy demands in the ipsilateral temporo-limbic areas, as determined by the “network control theory”, which models dynamic processes such as maintenance and transition between different mental states [53]. Patients with TLE require greater energy control compared to healthy controls in the anterior and mesial temporal areas of the seizure focus. These are also the areas of disrupted structural integrity (determined by MRI) and local glucose hypometabolism (demonstrated by FDG-PET). Thus, the substrates of brain dysfunction seem to be related to structural network aberrations and volume loss, on the one hand, and energetic inefficiency and hypometabolism, on the other hand [53].

Most of the studies related to connectome aberrations in patients with epilepsy have been performed on adult patients. In one of the pioneering connectome studies on children aged 1–17 years with non-lesional epilepsy, brain connectomes were compared between those with and without cognitive impairment [54]. It has been shown that higher structural network average path length and lower global network efficiency are seen more frequently in patients with cognitive deficits. These results were not influenced by age of onset, duration of epilepsy, or number of antiseizure medications. The number of daily seizures, however, was independently associated with cognitive impairment. Alterations in whole-brain network organization may serve as imaging biomarkers of cognitive deficits in children with epilepsy [54]. The network alterations in children with focal epilepsies may take rather longer, as in a recent longitudinal study with a median interscan period of 1.15 years, in which no changes were demonstrated in global network properties [55]. Further studies correlating cognitive decline with progressive structural network aberrations should have longer observation periods; these will provide valuable information about the disease mechanisms and may help in determining the best timing for epilepsy surgery in individual patients to prevent further cognitive impairment and, at the same time, achieve seizure freedom.

## 5. Personalized Treatment Planning

Whole-brain structural connectome models help in the understanding of seizure generation and spread. Identifying key nodes of seizure propagation may help in planning surgical procedures in individual patients by simulating epilepsy surgery on virtual models [56].

Functional connectomic profiles may predict seizure outcomes in patients who undergo DBS of the anterior nucleus of the thalamus due to intractable seizures [57]. In a pilot study on 18 patients with focal drug-resistant epilepsy who underwent DBS of the anterior nucleus of thalamus, it was demonstrated that greater functional connectivity between the seizure foci and the DBS site correlated with more favorable seizure outcome [57].

In another large study, in an attempt to identify a neural network associated with DBS targets for epilepsy, seed-to-voxel connectivity maps, weighted by seizure outcome, were used [58]. Areas associated with DBS were identified in normative resting state scans of 1000 individuals. Here also, the anterior nucleus of the thalamus was pinpointed as a central hub of the network with the highest betweenness and closeness values as opposed to the centromedian nucleus of the thalamus, caudate nucleus, mammillothalamic tract, and hippocampus, which demonstrated average centrality values [58]. The most important cortical hub for DBS in epilepsy patients was the anterior cingulate cortex [58]. Different DBS targets share a common cortical–subcortical network, which determines the effect of DBS in epilepsy patients. Individual variations in this network may be utilized for a personalized approach when planning DBS in selected patients [58].

DBS has important potential also in treating generalized epilepsies. The specific IGE network, determined by the coordinate network mapping technique, is based on subtle structural and functional neuroimaging abnormalities associated with generalized epilepsies [59]. This network peaks in the centromedian nucleus of the thalamus, making it a therapeutic target for DBS. Indeed, the outcome in 20 IGE patients who underwent DBS of the median nucleus of the thalamus was favorable, as 90% of the patients had an over 50% reduction in seizure frequency [60].

Identification of “risky” locations for developing epilepsy is important for planning therapeutic strategies in patients with potentially epileptogenic lesions. In a large case–control study on patients with post-stroke epilepsy versus those with stroke but without epilepsy, lesion locations related to epilepsy were mapped to a specific brain network defined by negative functional connectivity to basal ganglia and cerebellum [13]. These findings can also be applied to lesion types other than stroke, as the findings of the core group (post-stroke lesions) were validated on four different cohorts. Irrespective of the type of the lesion, if it was connected to the identified network, the chances of developing epilepsy were high. This “risky” network was also positively connected to the known network of thalamic DBS sites that improve seizure control. Thus, brain stimulation strategies in individual patients can be guided by lesion location if it maps to a network associated with epilepsy.

## 6. Methodological Limitations

Research related to brain connectivity is a rapidly developing and promising field, which has, however, some methodological limitations. DTI, a common technique for mapping white matter tracts, has limitations related to spatial resolution. The voxel size in DTI scans can lead to partial volume effects, where multiple tissue types are included in a single voxel, complicating the interpretation of connectivity. For instance, in regions with crossing fibers, DTI may not accurately represent the orientation and integrity of individual tracts, leading to misrepresentations of connectivity [61].

While fMRI provides insights into brain activity, its temporal resolution is limited (typically around 2–3 s). This delay can obscure the dynamics of neural processes, particularly in fast-evolving cognitive tasks, and potentially lead to incomplete or misleading conclusions about functional connectivity [62].

The choice of parcellation scheme (how the brain is divided into regions of interest) can significantly influence connectivity results. Different schemes may yield varied results regarding the number of regions and how they interact. Coarse parcellation might overlook important sub-regional connectivity, while overly fine parcellation could lead to noise and overfitting in the data. This raises concerns about the validity and reproducibility of findings across studies [63].

Determining appropriate statistical thresholds for identifying significant connections can be problematic. Overly lenient thresholds may result in false positives, while overly strict thresholds can lead to false negatives. The use of different statistical correction methods (e.g., FDR vs. Bonferroni correction) can lead to different conclusions about the significance of observed connectivity patterns, complicating comparisons between studies [64].

Reproducibility is a significant issue in brain connectome research. Variability in sample sizes, methodologies, and preprocessing steps can lead to inconsistent results across studies [65].

## 7. Conclusions

In summary, brain connectome aberrations are frequently observed in patients with focal and generalized epilepsies, both in children and adults. The most studied syndrome with regard to altered brain wiring is, however, drug-resistant TLE. Network alterations affect both grey and white matter, deep brain nuclei, including those of the ascending reticular formation. These structures may be involved in a disorganized connectome associated with epilepsy. The risk of developing epilepsy can be determined by linking locations of structural brain lesions to specific brain networks.

Connectome characteristics in epilepsy patients may predict responses to antiseizure medications and epilepsy surgery. Cognitive deficits have also been directly linked to aberrant brain networks in epilepsy patients.

Brain connectomes are helpful in understanding seizure generation and propagation within existing networks. Therefore, individualized interventional strategies could be developed based on connectome characteristics.

Further research should utilize advanced analytical techniques, such as machine learning, larger and diverse participant groups, and longitudinal designs. Collaborative efforts for systematic and rigorous analysis of “big data” are essential for maximizing the potential of the innovative field of connectomics.

## Figures and Tables

**Table 1:** Main content of the review: discussed topics.

Types of epilepsy	Temporal lobe epilepsy; idiopathic generalized epilepsy
Types of connectomes	Structural; functional; effective connectivity; covariance network
Theoretical implications	Understanding mechanisms of epileptogenesis
Clinical implications	Predicting seizure outcomes; personalized treatment strategies; cognition

## Abbreviations

DTI	Diffusion tensor imaging
MRI	Magnetic resonance tomography
DBS	Deep brain stimulation
TLE	Temporal lobe epilepsy
IGE	Idiopathic generalized epilepsy
ENIGMA	Enhancing NeuroImaging Genetics through Meta-Analysis
ASM	Anti-seizure medication
CA	Cornu ammonis
ROC	Receiver operating characteristic
EEG	Electroencephalography
FDG-PET	Fluorodeoxyglucose positron emission tomography
IQ	Intelligence quotient

## References

[1] Sporns O. (2011). The human connectome: A complex network. Ann. N. Y. Acad. Sci..

[2] Bullmore E., Sporns O. (2012). The economy of brain network organization. Nat. Rev. Neurosci..

[3] Biswal B.B., Mennes M., Zuo X.N., Gohel S., Kelly C., Smith S.M., Beckmann C.F., Adelstein J.S., Buckner R.L., Colcombe S. (2010). Toward discovery science of human brain function. Proc. Natl. Acad. Sci. USA.

[4] Glasser M.F., Coalson T.S., Robinson E.C., Hacker C.D., Harwell J., Yacoub E., Ugurbil K., Andersson J., Beckmann C.F., Jenkinson M. (2016). A multi-modal parcellation of human cerebral cortex. Nature.

[5] Hagmann P., Cammoun L., Gigandet X., Meuli R., Honey C.J., Wedeen V.J., Sporns O. (2008). Mapping the structural core of human cerebral cortex. PLoS Biol..

[6] Royer J., Bernhardt B.C., Lariviere S., Gleichgerrcht E., Vorderwulbecke B.J., Vulliemoz S., Bonilha L. (2022). Epilepsy and brain network hubs. Epilepsia.

[7] Bernhardt B.C., Bonilha L., Gross D.W. (2015). Network analysis for a network disorder: The emerging role of graph theory in the study of epilepsy. Epilepsy Behav..

[8] Engel J., Thompson P.M., Stern J.M., Staba R.J., Bragin A., Mody I. (2013). Connectomics and epilepsy. Curr. Opin. Neurol..

[9] Gleichgerrcht E., Kocher M., Bonilha L. (2015). Connectomics and graph theory analyses: Novel insights into network abnormalities in epilepsy. Epilepsia.

[10] Sohn J. (2023). Synaptic configuration and reconfiguration in the neocortex are spatiotemporally selective. Anat. Sci. Int..

[11] Royer J., Lariviere S., Rodriguez-Cruces R., Cabalo D.G., Tavakol S., Auer H., Ngo A., Park B.Y., Paquola C., Smallwood J. (2023). Cortical microstructural gradients capture memory network reorganization in temporal lobe epilepsy. Brain.

[12] Van Essen D.C., Smith S.M., Barch D.M., Behrens T.E., Yacoub E., Ugurbil K., WU-Minn HCP Consortium (2013). The WU-Minn Human Connectome Project: An overview. Neuroimage.

[13] Schaper F., Nordberg J., Cohen A.L., Lin C., Hsu J., Horn A., Ferguson M.A., Siddiqi S.H., Drew W., Soussand L. (2023). Mapping Lesion-Related Epilepsy to a Human Brain Network. JAMA Neurol..

[14] Kramer M.A., Cash S.S. (2012). Epilepsy as a disorder of cortical network organization. Neuroscientist.

[15] Blumcke I., Thom M., Wiestler O.D. (2002). Ammon’s horn sclerosis: A maldevelopmental disorder associated with temporal lobe epilepsy. Brain Pathol..

[16] Voets N.L., Bernhardt B.C., Kim H., Yoon U., Bernasconi N. (2011). Increased temporolimbic cortical folding complexity in temporal lobe epilepsy. Neurology.

[17] Bernhardt B.C., Chen Z., He Y., Evans A.C., Bernasconi N. (2011). Graph-theoretical analysis reveals disrupted small-world organization of cortical thickness correlation networks in temporal lobe epilepsy. Cereb. Cortex.

[18] Bernhardt B.C., Bernasconi N., Kim H., Bernasconi A. (2012). Mapping thalamocortical network pathology in temporal lobe epilepsy. Neurology.

[19] Lariviere S., Weng Y., Vos de Wael R., Royer J., Frauscher B., Wang Z., Bernasconi A., Bernasconi N., Schrader D.V., Zhang Z. (2020). Functional connectome contractions in temporal lobe epilepsy: Microstructural underpinnings and predictors of surgical outcome. Epilepsia.

[20] Javidi S.S., He X., Ankeeta A., Zhang Q., Citro S., Sperling M.R., Tracy J.I. (2024). Edge-wise analysis reveals white matter connectivity associated with focal to bilateral tonic-clonic seizures. Epilepsia.

[21] Xie K., Royer J., Rodriguez-Cruces R., Horwood L., Ngo A., Arafat T., Auer H., Sahlas E., Chen J., Zhou Y. (2025). Temporal Lobe Epilepsy Perturbs the Brain-Wide Excitation-Inhibition Balance: Associations with Microcircuit Organization, Clinical Parameters, and Cognitive Dysfunction. Adv. Sci..

[22] Weng Y., Lariviere S., Caciagli L., Vos de Wael R., Rodriguez-Cruces R., Royer J., Xu Q., Bernasconi N., Bernasconi A., Thomas Yeo B.T. (2020). Macroscale and microcircuit dissociation of focal and generalized human epilepsies. Commun. Biol..

[23] Wang Z., Lariviere S., Xu Q., Vos de Wael R., Hong S.J., Wang Z., Xu Y., Zhu B., Bernasconi N., Bernasconi A. (2019). Community-informed connectomics of the thalamocortical system in generalized epilepsy. Neurology.

[24] Klamer S., Ethofer T., Torner F., Sahib A.K., Elshahabi A., Marquetand J., Martin P., Lerche H., Erb M., Focke N.K. (2018). Unravelling the brain networks driving spike-wave discharges in genetic generalized epilepsy-common patterns and individual differences. Epilepsia Open.

[25] Li Hegner Y., Marquetand J., Elshahabi A., Klamer S., Lerche H., Braun C., Focke N.K. (2018). Increased Functional MEG Connectivity as a Hallmark of MRI-Negative Focal and Generalized Epilepsy. Brain Topogr..

[26] Lariviere S., Royer J., Rodriguez-Cruces R., Paquola C., Caligiuri M.E., Gambardella A., Concha L., Keller S.S., Cendes F., Yasuda C.L. (2022). Structural network alterations in focal and generalized epilepsy assessed in a worldwide ENIGMA study follow axes of epilepsy risk gene expression. Nat. Commun..

[27] Bayrak S., de Wael R.V., Schaare H.L., Hettwer M.D., Caldairou B., Bernasconi A., Bernasconi N., Bernhardt B.C., Valk S.L. (2022). Heritability of hippocampal functional and microstructural organisation. Neuroimage.

[28] Engel J. (2001). Mesial temporal lobe epilepsy: What have we learned?. Neuroscientist.

[29] Wiebe S., Blume W.T., Girvin J.P., Eliasziw M. (2001). Effectiveness and Efficiency of Surgery for Temporal Lobe Epilepsy Study Group. A randomized, controlled trial of surgery for temporal-lobe epilepsy. N. Engl. J. Med..

[30] Spencer M.D., Chura L.R., Holt R.J., Suckling J., Calder A.J., Bullmore E.T., Baron-Cohen S. (2012). Failure to deactivate the default mode network indicates a possible endophenotype of autism. Mol. Autism.

[31] Tellez-Zenteno J.F., Dhar R., Wiebe S. (2005). Long-term seizure outcomes following epilepsy surgery: A systematic review and meta-analysis. Brain.

[32] de Tisi J., Bell G.S., Peacock J.L., McEvoy A.W., Harkness W.F., Sander J.W., Duncan J.S. (2011). The long-term outcome of adult epilepsy surgery, patterns of seizure remission, and relapse: A cohort study. Lancet.

[33] Morgan V.L., Sainburg L.E., Johnson G.W., Janson A., Levine K.K., Rogers B.P., Chang C., Englot D.J. (2022). Presurgical temporal lobe epilepsy connectome fingerprint for seizure outcome prediction. Brain Commun..

[34] Bernhardt B.C., Fadaie F., Liu M., Caldairou B., Gu S., Jefferies E., Smallwood J., Bassett D.S., Bernasconi A., Bernasconi N. (2019). Temporal lobe epilepsy: Hippocampal pathology modulates connectome topology and controllability. Neurology.

[35] Girardi-Schappo M., Fadaie F., Lee H.M., Caldairou B., Sziklas V., Crane J., Bernhardt B.C., Bernasconi A., Bernasconi N. (2021). Altered communication dynamics reflect cognitive deficits in temporal lobe epilepsy. Epilepsia.

[36] Keller S.S. (2022). Fingerprinting seizure outcome after temporal lobe surgery using preoperative connectomic mapping. Brain Commun..

[37] Lin Q., Cao D., Li W., Zhang Y., Li Y., Liu P., Huang X., Huang K., Gong Q., Zhou D. (2025). Connectome architecture for gray matter atrophy and surgical outcomes in temporal lobe epilepsy. Epilepsia.

[38] Gleichgerrcht E., Keller S.S., Drane D.L., Munsell B.C., Davis K.A., Kaestner E., Weber B., Krantz S., Vandergrift W.A., Edwards J.C. (2020). Temporal Lobe Epilepsy Surgical Outcomes Can Be Inferred Based on Structural Connectome Hubs: A Machine Learning Study. Ann. Neurol..

[39] Bonilha L., Jensen J.H., Baker N., Breedlove J., Nesland T., Lin J.J., Drane D.L., Saindane A.M., Binder J.R., Kuzniecky R.I. (2015). The brain connectome as a personalized biomarker of seizure outcomes after temporal lobectomy. Neurology.

[40] He X., Doucet G.E., Pustina D., Sperling M.R., Sharan A.D., Tracy J.I. (2017). Presurgical thalamic “hubness” predicts surgical outcome in temporal lobe epilepsy. Neurology.

[41] Sinha N., Wang Y., Moreira da Silva N., Miserocchi A., McEvoy A.W., de Tisi J., Vos S.B., Winston G.P., Duncan J.S., Taylor P.N. (2021). Structural Brain Network Abnormalities and the Probability of Seizure Recurrence After Epilepsy Surgery. Neurology.

[42] Morgan V.L., Englot D.J., Rogers B.P., Landman B.A., Cakir A., Abou-Khalil B.W., Anderson A.W. (2017). Magnetic resonance imaging connectivity for the prediction of seizure outcome in temporal lobe epilepsy. Epilepsia.

[43] DeSalvo M.N., Tanaka N., Douw L., Cole A.J., Stufflebeam S.M. (2020). Contralateral Preoperative Resting-State Functional MRI Network Integration Is Associated with Surgical Outcome in Temporal Lobe Epilepsy. Radiology.

[44] Rodriguez-Cruces R., Velazquez-Perez L., Rodriguez-Leyva I., Velasco A.L., Trejo-Martinez D., Barragan-Campos H.M., Camacho-Tellez V., Concha L. (2018). Association of white matter diffusion characteristics and cognitive deficits in temporal lobe epilepsy. Epilepsy Behav..

[45] Balachandra A.R., Kaestner E., Bahrami N., Reyes A., Lalani S., Macari A.C., Paul B.M., Bonilha L., McDonald C.R. (2020). Clinical utility of structural connectomics in predicting memory in temporal lobe epilepsy. Neurology.

[46] Kaestner E., Balachandra A.R., Bahrami N., Reyes A., Lalani S.J., Macari A.C., Voets N.L., Drane D.L., Paul B.M., Bonilha L. (2020). The white matter connectome as an individualized biomarker of language impairment in temporal lobe epilepsy. Neuroimage Clin..

[47] Fadaie F., Lee H.M., Caldairou B., Gill R.S., Sziklas V., Crane J., Bernhardt B.C., Hong S.J., Bernasconi A., Bernasconi N. (2021). Atypical functional connectome hierarchy impacts cognition in temporal lobe epilepsy. Epilepsia.

[48] Englot D.J., Gonzalez H.F.J., Reynolds B.B., Konrad P.E., Jacobs M.L., Gore J.C., Landman B.A., Morgan V.L. (2018). Relating structural and functional brainstem connectivity to disease measures in epilepsy. Neurology.

[49] Englot D.J., D’Haese P.F., Konrad P.E., Jacobs M.L., Gore J.C., Abou-Khalil B.W., Morgan V.L. (2017). Functional connectivity disturbances of the ascending reticular activating system in temporal lobe epilepsy. J. Neurol. Neurosurg. Psychiatry.

[50] Xie K., Royer J., Lariviere S., Rodriguez-Cruces R., Frassle S., Cabalo D.G., Ngo A., DeKraker J., Auer H., Tavakol S. (2023). Atypical connectome topography and signal flow in temporal lobe epilepsy. bioRxiv.

[51] Zhang Q., Hudgins S., Struck A.F., Ankeeta A., Javidi S.S., Sperling M.R., Hermann B.P., Tracy J.I. (2024). Association of Normative and Non-Normative Brain Networks with Cognitive Function in Patients with Temporal Lobe Epilepsy. Neurology.

[52] He X., Bassett D.S., Chaitanya G., Sperling M.R., Kozlowski L., Tracy J.I. (2018). Disrupted dynamic network reconfiguration of the language system in temporal lobe epilepsy. Brain.

[53] He X., Caciagli L., Parkes L., Stiso J., Karrer T.M., Kim J.Z., Lu Z., Menara T., Pasqualetti F., Sperling M.R. (2022). Uncovering the biological basis of control energy: Structural and metabolic correlates of energy inefficiency in temporal lobe epilepsy. Sci. Adv..

[54] Woodfield J., Chin R.F.M., van Schooneveld M.M.J., van den Heuvel M., Bastin M.E., Braun K.P.J. (2023). The association of structural connectome efficiency with cognition in children with epilepsy. Epilepsy Behav..

[55] Chari A., Piper R.J., Wilson-Jeffers R., Ruiz-Perez M., Seunarine K., Tahir M.Z., Clark C.A., Rosch R., Scott R.C., Baldeweg T. (2025). Longitudinal alterations in brain networks and thalamocortical connectivity in paediatric focal epilepsy: A structural connectomics pilot study. Brain Commun..

[56] Wang C., Chen S., Huang L., Yu L. (2022). Prediction and control of focal seizure spread: Random walk with restart on heterogeneous brain networks. Phys. Rev. E.

[57] Xu C., Qi L., Wang X., Schaper F., Wu D., Yu T., Yan X., Jin G., Wang Q., Wang X. (2023). Functional connectomic profile correlates with effective anterior thalamic stimulation for refractory epilepsy. Brain Stimul..

[58] Vetkas A., Germann J., Elias G., Loh A., Boutet A., Yamamoto K., Sarica C., Samuel N., Milano V., Fomenko A. (2022). Identifying the neural network for neuromodulation in epilepsy through connectomics and graphs. Brain Commun..

[59] Ji G.J., Fox M.D., Morton-Dutton M., Wang Y., Sun J., Hu P., Chen X., Jiang Y., Zhu C., Tian Y. (2025). A generalized epilepsy network derived from brain abnormalities and deep brain stimulation. Nat. Commun..

[60] Cukiert A., Cukiert C.M., Burattini J.A., Mariani P.P. (2020). Seizure outcome during bilateral, continuous, thalamic centromedian nuclei deep brain stimulation in patients with generalized epilepsy: A prospective, open-label study. Seizure.

[61] Jones D.K., Leemans A. (2011). Diffusion tensor imaging. Methods Mol. Biol..

[62] Huettel S.A., Song A.W., McCarthy G. (2009). Functional Magnetic Resonance Imaging.

[63] Bryce N.V., Flournoy J.C., Guassi Moreira J.F., Rosen M.L., Sambook K.A., Mair P., McLaughlin K.A. (2021). Brain parcellation selection: An overlooked decision point with meaningful effects on individual differences in resting-state functional connectivity. Neuroimage.

[64] Eklund A., Nichols T.E., Knutsson H. (2016). Cluster failure: Why fMRI inferences for spatial extent have inflated false-positive rates. Proc. Natl. Acad. Sci. USA.

[65] Poldrack R.A., Yarkoni T. (2016). From Brain Maps to Cognitive Ontologies: Informatics and the Search for Mental Structure. Annu. Rev. Psychol..

